# Vitamin D_3_ Deficiency Differentially Affects Functional and Disease Outcomes in the G93A Mouse Model of Amyotrophic Lateral Sclerosis

**DOI:** 10.1371/journal.pone.0029354

**Published:** 2011-12-27

**Authors:** Jesse A. Solomon, Alexandro Gianforcaro, Mazen J. Hamadeh

**Affiliations:** 1 School of Kinesiology and Health Science, Faculty of Health, York University, Toronto, Ontario, Canada; 2 Muscle Health Research Centre, York University, Toronto, Ontario, Canada; 3 Department of Pediatrics, McMaster University, Hamilton, Ontario, Canada; Washington University, United States of America

## Abstract

Amyotrophic lateral sclerosis (ALS) is a neuromuscular disease characterized by motor neuron death in the central nervous system. Vitamin D supplementation increases antioxidant activity, reduces inflammation and improves motor neuron survival. We have previously demonstrated that vitamin D_3_ supplementation at 10× the adequate intake improves functional outcomes in a mouse model of ALS.

**Objective:**

To determine whether vitamin D deficiency influences functional and disease outcomes in a mouse model of ALS.

**Methods:**

At age 25 d, 102 G93A mice (56 M, 46 F) were divided into two vitamin D_3_ groups: 1) adequate (AI; 1 IU D_3_/g feed) and 2) deficient (DEF; 0.025 IU D_3_/g feed). At age 113 d, *tibialis anterior* (TA), *quadriceps* (quads) and brain were harvested from 42 mice (22 M and 20 F), whereas the remaining 60 mice (34 M and 26 F) were followed to endpoint.

**Results:**

During disease progression, DEF mice had 25% (P = 0.022) lower paw grip endurance AUC and 19% (P = 0.017) lower motor performance AUC vs. AI mice. Prior to disease onset (CS 2), DEF mice had 36% (P = 0.016) lower clinical score (CS) vs. AI mice. DEF mice reached CS 2 six days later vs. AI mice (P = 0.004), confirmed by a logrank test which revealed that DEF mice reached CS 2 at a 43% slower rate vs. AI mice (HR = 0.57; 95% CI: 0.38, 1.74; P = 0.002). Body weight-adjusted TA (AI: r = 0.662, P = 0.001; DEF: r = 0.622, P = 0.006) and quads (AI: r = 0.661, P = 0.001; DEF: r = 0.768; P<0.001) weights were strongly correlated with age at CS 2.

**Conclusion:**

Vitamin D_3_ deficiency improves early disease severity and delays disease onset, but reduces performance in functional outcomes following disease onset, in the high-copy G93A mouse.

## Introduction

Amyotrophic lateral sclerosis (ALS) is a neuromuscular disease characterized by motor neuron atrophy and death in the brain and spinal cord [1]–[3]. Symptoms of the disease begin with muscle weakness, ultimately leading to complete paralysis and death resulting from respiratory failure [4]. Survival is typically 2–4 years from diagnosis [5]. To date, Riluzole remains the most effective pharmaceutical for the treatment of ALS [6]. In clinical studies, Riluzole improves survival by ∼2 months [7], whereas functional benefits are equivocal [6], [8]. Transgenic mice that overexpress the mutant (G93A) human Cu/Zn-superoxide dismutase gene follow a similar disease pattern as ALS patients and thus serve as an animal model for ALS [9]. The pathophysiology of ALS is complex and includes glutamate excitotoxicity, oxidative stress, inflammation, mitochondrial dysfunction, protein misfolding and aggregation, and apoptosis [10].

Several nutrition intervention studies have attempted to influence one or more pathophysiologies of ALS, as described by Patel and Hamadeh [10]. In animal models of ALS, anti-glutamatergic agents such as branched chain amino acids, L-threonine and magnesium have yielded modest and conflicting results. However, a delay in disease onset and/or an extended lifespan was observed for treatments using antioxidants (5–26% and 4–60%, respectively; Ginkgo biloba, melatonin, lyophilized red wine, co-enzyme Q10, N-acetyl cysteine, catalase, ginseng, folic acid and vitamin B12, epigallocatechin gallate, L-carnitine, vitamin E and trientine plus ascorbate), as well as therapies targeting mitochondrial dysfunction (7% and 6–17%, respectively; creatine and pyruvate) and SOD1 stability (8% and 5–10%, respectively; trientine (alone and with ascorbate) and zinc). Dietary modifications such as high fat and fast food diets extended lifespan by 20–22%, however caloric restriction hastened disease onset and endpoint in the G93A mouse model [11]–[13].

Vitamin D is a secosteroid hormone obtained from skin exposure to UVB radiation (290–320 nm). Vitamin D is found naturally in deep ocean fish and fortified in foods such as margarine, milk and cereals [14]–[16]. Synthesis of vitamin D_3_ from sun exposure is dependent on the zenith angle of the sun, season, time of day and skin type [17]–[19]. Vitamin D is best known for its role in calcium and phosphorous homeostasis, however the vitamin D receptor (VDR) is present in cells throughout the body and influences cell proliferation and differentiation as well as immunomodulation [20]. The VDR has been found in tissues such as brain [21], spinal cord [22], heart [23], breast [24], pancreas [25], small intestine [26], and skeletal muscle [27], however there is controversy in the literature as one recent study has shown that the VDR is not present in muscle [28]. In addition, the presence of 25-hydroxyvitamin D_3_-1α-hydroxylase, the enzyme that converts calcidiol (used to assess vitamin D status) to calcitriol (most active form of vitamin D) [20], is also present in most tissues in the body including the brain [29], spinal cord [30], pancreas [29], kidneys [31], colon [29], lymph nodes [29], skin [29] and smooth muscle [32].

Vitamin D influences several pathophysiologies of ALS including glutamate excitotoxicity, oxidative stress, inflammation and apoptosis. *In vitro*, Taniura et al [33] pretreated cultured rat cortical neurons with calcitriol followed by a glutamate insult. Neuron survival was ∼17% higher when treated with 10 nM calcitriol and ∼50% higher with 100 nM compared to a calcitriol-free control. Vitamin D also reduces oxidative stress and regulates the inflammatory response by down regulating the expression of pro-inflammatory cytokines and increasing gene expression of anti-inflammatory cytokines (i.e. IL-10) [34]. In one study using a rodent model of diabetes, calcitriol supplementation (5,000 IU/kg b.wt./d) for 4 wk prior to diabetes induction increased the antioxidant activity of superoxide dismutase (liver ∼2.9 fold and kidney ∼4.2 fold), glutathione peroxidase (liver and kidney ∼2.2 fold) and catalase (liver ∼2 fold and kidney ∼3.5 fold) compared to diabetic rat controls [35]. *In vitro*, high levels of calcitriol inhibit nuclear factor kappa B (NF-kB), a transcription factor which increases inducible nitric oxide expression, ROS and pro-inflammatory cytokines such as interleukin-1β (IL-1β), IL-2, IL-6, IL-8, and tumor necrosis factor-α (TNF-α) [34], [36], [37]. *In vivo*, using a rat model of cortical infarction, 8 day pretreatment with 1 µg/kg/d calcitriol increased glial cell line derived neurotrophic factor (GDNF) by 2 fold [38] compared to a calcitriol-free control. GDNF promotes motor neuron survival *in vitro* [39].

Observational studies have linked low vitamin D status to neurological, autoimmune and inflammatory diseases that share some common pathophysiologies with ALS. In one study, men and women diagnosed with Parkinson's and Alzheimer's disease had 14% and 6% lower mean plasma calcidiol concentrations compared to controls, respectively (79.8 nmol/L and 87.0 nmol/L vs. 92.5 nmol/L, respectively) [40]. Moreover, 55% of Parkinson's disease patients and 41% of Alzheimer's disease patients had serum calcidiol ≤75 nmol/L compared to 36% of controls. *In vitro*, calcitriol decreased apoptosis and L-type calcium channel (LVSCC A1C) mRNA in rat neuronal cultures exposed to amyloid-β (Aβ) [41]. Corrected for control (no Aβ or calcitriol added), pretreatment of calcitriol followed by administration of Aβ decreased cytotoxicity by ∼2.5 fold compared to Aβ alone at 72 hours. As well, caspase 3 protein was lower in calcitriol-treated cultures exposed to Aβ compared to cultures treated with Aβ alone.

IL-10 KO mice, a model of inflammatory bowel disease, given a vitamin D deficient diet had 41% lower body weight compared to mice supplemented with calcitriol (0.005 µg/d), indicating severe tissue wasting. Moreover, after correcting for body weight, vitamin D deficient mice had 83% heavier small intestines, used as a measure of disease severity and inflammation, compared to mice supplemented with calcitriol [42]. Using a mouse model of arthritis, VDR KO mice had an earlier disease onset and more severe symptoms of disease [43]. At 9 wk of age, VDR KO mice had 47% greater grip strength loss, 91% greater paw swelling, 83% higher proteoglycan loss, 35% higher chondrocyte death and 2 fold higher bone erosion compared to controls. Immunohistochemical analysis revealed a 2 fold higher concentration of synovial macrophages in the hind paws of VDR KO mice compared to controls.

In a mouse model of multiple sclerosis (experimental autoimmune encephalomyelitis; EAE), disease was prevented with an oral dose of 20 ng/day of calcitriol prior to disease induction, whereas mice fed a diet devoid of vitamin D followed a typical EAE disease progression [44]. Calcitriol administered at early signs of overt EAE symptoms halted disease progression. The effects of calcitriol were reversible. When calcitriol was removed from the diet, mice resumed EAE disease progression, reaching a disease severity equal to mock-treated controls. Pups born from vitamin D deficient mothers and given a vitamin D deficient diet post-weaning reached EAE disease onset 43% earlier compared to offspring supplemented with 20 ng/day of calcitriol [44]. Interestingly, in a different study using the same animal model, pups born from vitamin D deficient mothers, but given a diet containing 1,500 IU vitamin D_3_/kg feed beginning at 4 wk of age, had a delay in disease onset by 17–34% and 61% lower peak disease severity compared to pups born from vitamin D replete mothers and fed a diet containing 1,500 IU vitamin D_3_/kg feed at 4 wk of age [45]. Also, in the same animal model, second generation vitamin D deficient mice (fed a diet devoid of vitamin D) had a delay in disease onset (∼49–58%) and lower disease severity (41–55%) compared to EAE control mice [46].

Human epidemiological studies have linked low vitamin D intake with type 1 diabetes [47] and low serum calcidiol concentrations with an elevated risk for cardiovascular disease [48], atherosclerosis [49], type 2 diabetes [50] and breast cancer [51]. There is controversy surrounding the optimal vitamin D dosage as studies have shown a positive [52], negative [53], U-shaped [54] and no [55] relationship between serum calcidiol and risk of prostate cancer. Furthermore, one study found a negative relationship between vitamin D intake and risk of pancreatic cancer [56], whereas another study found a positive relationship between serum calcidiol and pancreatic cancer risk [57].

We have previously demonstrated that vitamin D_3_ supplementation at 10× the rodent AI attenuates the decline in paw grip endurance and motor performance in the G93A mouse model of ALS [58]. We were interested in ascertaining whether dietary vitamin D_3_ restriction would reverse these observations in the same disease model. Hence, the objective of this study was to investigate the effects of vitamin D deficiency on functional and disease outcomes, disease onset and lifespan in the G93A mouse model of ALS.

## Methods

### Ethics Statement

The study involved the use of rodents. The experimental protocol strictly followed guidelines put forth by the Canadian Council of Animal Care and York University Animal Research Ethics Board. All necessary steps were taken to ameliorate suffering to animals involved in the study. All procedures were approved by the York University Animal Research Ethics Board (protocol # 2007-9).

### Animals

Male B6SJL-TgN(SOD1-G93A)1Gur hemizygous mice (No. 002726) were harem-bred with nonaffected female B6SJL control mice (No. 100012; Jackson Laboratory, Bar Harbor, ME). Breeding mice consumed Research Diet AIN-93G (1 IU D_3_/g feed; Research Diet, New Brunswick, NJ). The presence of the human G93A transgene was confirmed using polymerase chain reaction (PCR) amplification of DNA extracted from ear samples as outlined by Sigma-Aldrich (XNAT REDExtract-N-Amp Tissue PCR Kit; XNAT-1KT). All animals were housed individually at age 25 d in a 12 h light/dark cycle.

### Study design

One hundred and two G93A mice (46 F, 56 M) were fed a diet containing an adequate amount of vitamin D_3_ (1 IU/g feed; Research Diet AIN-93G; Product # D10012G; Research Diets Inc, New Brunswick, NJ) *ad libitum* after weaning (21 d) until the study commenced at age 25 d. At age 25 d, mice were housed in individual cages and divided into one of two groups: 1) adequate vitamin D_3_ (AI; 1 IU D_3_/g feed; 23 F, 31 M; Research Diet AIN-93G) and 2) deficient vitamin D_3_ (DEF; 0.025 IU D_3_/g feed; 23 F, 25 M; Product # D10030801; Research Diets Inc, New Brunswick, NJ; Table 1) [59], [60]. Of these mice, 23 AI (11 F, 12 M) and 19 DEF (9 F, 10 M) were sacrificed at age 113 d for *tibialis anterior*, *quadriceps* and brain harvesting, whereas the remaining mice (n = 60) were followed to endpoint. The age of sacrifice (113 d) was based on our preliminary results showing differences in clinical score (CS) between DEF and AI mice at age 113 d. For tissue harvesting, *tibialis anterior*, *quadriceps* and brain were weighed immediately after removal then flash frozen in liquid nitrogen.

**Table 1:** Macro- and micro-nutrient content of the adequate intake (AI) vitamin D_3_ and deficient (DEF) vitamin D_3_ diets.

Nutrient	Diet	
	AI	DEF
Energy (kcal/g)	4	4
Carbohydrate (%)	64	64
Protein (%)	20	20
Fat (%)	7	7
Vitamin D_3_ (IU/g)	1^a^	0.025^b^
Calcium (%)	0.5^c^	0.5^c^
Vitamin mix V10037 (mg/g)	10	0
Vitamin mix V13203 (mg/g)	0	10
Mineral mix S100022G (mg/g)	35	35

Diets provided by Research Diets (AIN-93G; New Brunswick, NJ).

a , included in vitamin mix V10037 [59].

b , included in vitamin mix V13203.

c , included in mineral mix S100022G [60].

When mice attained a CS of 3.0, food and calorie-free gel (Harlan-Gel, Harlan Teklad, Madison, WI) were placed on the floor of the cage to fulfill the requirements of the ethics committee. The calorie-free gel contained synthetic polymers (WATER LOCK® superabsorbent polymer G-400, G- 430, G-500, G-530; 95% by weight) and methanol (4.5% by weight). All measurements were conducted by two researchers who were blinded to the diets. Following statistical analysis, researchers were unblinded. Intra-tester coefficients of variation (CV) were 0.0% for ability to move and 1.2% for clinical score for researcher #1, and 0.0% for ability to move and 1.1% for clinical score for researcher #2. The inter-researcher CV are 1.06% for ability to move and 0.96% for clinical score.

### Food intake

Food intake measurements began at age 25 d and were recorded twice per wk for all mice until endpoint.

### Body weight

Beginning at age 25 d, body weight measurements were recorded twice per wk until mice reached a clinical score of 3.0, thereafter measurements were recorded daily.

### Ability to move

Beginning at age 60 d, ability to move was assessed twice per wk until mice reached a clinical score of 3.0, thereafter it was assessed daily. Ability to move followed a 5-point scale: 4 = normal mobility, 3 = moving with limited use of the hindlimbs, 2 = moving with the use of the forelimbs, 1 = moving only for a short period with the use of the forelimbs, and 0 = not moving.

### Paw grip endurance

Beginning at age 60 d, paw grip endurance (PaGE) was measured 3 times every 10 d (measurement days were separated by 2–3 days), using the modified hanging wire test [61], [62]. Animals were placed on a wire grid held at a height of ∼40 cm, the grid was gently shaken to cause the mouse to fasten its grip on the wires then inverted. The time was recorded until the mouse lost its grip, for a maximum score of 180 s. This test was completed in triplicate, with the highest score used for analysis.

### Motor performance

Beginning at age 60 d, motor performance was measured once every 10 d using the rotarod test (AccuScan Instruments, Inc., Columbus, OH). Mice were placed on a rod (30 mm diameter at a height of 39 cm, covered with corrugated rubber to allow for traction) that rotated at a gradually increasing speed to 45 rpm over 60 s and remained at 45 rpm until the mouse could no longer stay on the rod. The rotarod apparatus was interfaced with a computer that initiated the test and recorded the competency score. Motion sensors placed at the bottom of the rotarod chamber were activated when the mouse fell off the rod and, as a result, the computer ended the recording session. The test was performed in triplicate, with the highest score used for analysis.

### Clinical score

Beginning at age 60 d, clinical score was assessed daily until endpoint and followed an 8-point scale based on signs of weakness exhibited by the mice in order to establish disease severity: 0 = no evidence of disease, 1 = shaking or splaying of the hindlimbs when suspended by the tail (an indication of weakness in the hindlimbs), 1.5 = weakness in one hindlimb (compensation for footdrop), 2 = weakness in both hindlimbs (change in gait; used as disease onset when attained on two consecutive days), 2.5 = extreme weakness in one hindlimb (inability to dorsiflex), 3 = extreme weakness in both hindlimbs, 3.5 = functional paralysis in one hindlimb, 4 = functional paralysis in both hindlimbs, and 5 = mouse cannot right itself within 20 s after being placed on its side (considered as endpoint) [63]. For all mice, clinical score was assessed prior to all other functional measurements.

### *Tibialis anterior, quadriceps* and brain

Forty-two G93A mice were sacrificed at age 113 d, whereas the rest of the mice (n = 60) were followed to endpoint. Mice were anesthetized with isoflurane gas and maintained under general anesthesia as tissues were collected. *Tibialis anterior, quadriceps* and brain were removed and immediately weighed.

### Statistical analysis

A three-way repeated measures ANOVA (between-subject factors: diet and sex; within-subject factor: time) was used to determine significant sex differences over time in food intake, food intake corrected for body weight, body weight, ability to move, PaGE, motor performance and clinical score. A two-way repeated measures ANOVA (between-subject factor: diet; within-subject factor: time) was used to determine significant differences over time for ability to move, PaGE, motor performance and clinical score in four ways: 1) using data from the first day of testing (age 60 d) until the age at which the first group achieved a mean clinical score of 5 (endpoint; 141 d), 2) using data from the first day of testing (age 60 d) until the age at which the first group achieved a mean clinical score of 2 (disease onset; 105 d), 3) using data from the first day of testing (age 60 d) until age 113 d (age at which 42 mice were harvested for *tibialis anterior*, *quadriceps* and brain tissue), and 4) using data from the age at which the first group achieved a mean clinical score of 2 (disease onset; 105 d) until the age at which the first group achieved a mean clinical score of 5 (endpoint; 141 d) (i.e. during disease progression). Statistical analysis of 60–113 d was chosen to determine if an additional 8 days of data would influence the results from 60–105 d. Statistical analysis using data from 60–105 d with and without harvest mice as well as 60–113 d with and without harvest mice were not different, therefore the results reported are for 60–105 d without harvest mice; age at CS 2 and CS 4 were included when determining the effect of diet on age at disease onset and hindlimb paralysis, respectively. When ANOVA indicated significance, a Newman-Keuls post-hoc test was used to determine the source of difference. A Student's t-test was used to determine significant differences in disease onset (CS 2), hindlimb paralysis (CS 4), endpoint (CS 5), number of days between CS 2 to CS 5 (disease progression; DISPRO), area under the curve (AUC) and cumulative scores between the diets (within sexes and sexes combined). Statistical analyses for AUC and cumulative scores for ability to move, PaGE, motor performance and clinical score were conducted in three ways: 1) using data from the first day of testing (age 60 d) until individual endpoint (CS 5) specific for each mouse, 2) using data from the first day of testing (age 60 d) until individual disease onset (CS 2) specific for each mouse, and 3) using data from individual disease onset (CS 2) until individual endpoint (CS 5) specific for each mouse (i.e. during disease progression). AUC and cumulative scores for each mouse were corrected for the number of days between the first day of testing (age 60 d) and endpoint (first approach), between the first day of testing (age 60 d) and disease onset (second approach), or between disease onset and endpoint (third approach, i.e. during disease progression). Analysis of data between disease onset and endpoint was performed to specifically investigate the effect of the DEF diet on disease progression. A logrank test was used to determine differences in the rate at which the groups reached disease onset, hindlimb paralysis and endpoint.

The following correlations were performed: ability to move, PaGE and motor performance vs. CS; PaGE and motor performance vs. ability to move; as well as motor performance vs. PaGE. The above correlations were analyzed in four ways: 1) a three-way repeated measures ANOVA (between-subject factors: diet and sex; within-subject factor: repeated measures) to identify sex differences, 2) a two-way repeated measures ANOVA (diet and repeated measures) to identify diet differences within sex or when sexes were combined, 3) a two-way ANOVA (diet and sex) to identify sex differences in AUC and 4) a Student's t-test to identify diet differences in AUC within sex or when sexes were combined.

A Student's t-test was used to determine diet and sex differences in absolute and body weight-adjusted *tibialis anterior*, *quadriceps* and brain weights. Body weight-adjusted *tibialis anterior*, *quadriceps* and brain weights were correlated with age at CS 2 for each harvest mouse. Group means for body weight-adjusted *tibialis anterior*, *quadriceps* and brain weights were also correlated with the group means for age at CS 4 and CS 5, as well as DISPRO, for non-harvest mice within the same diet and sex. A two-tailed test was used for all statistical comparisons for outcome measures except for ability to move, PaGE and motor performance where a one-tailed test was used, because based on the scientific literature we *a priori* hypothesized that vitamin D deficiency would be detrimental to functional outcomes (ability to move, PaGE and motor performance). Our lab has recently demonstrated that vitamin D_3_ supplementation at 10 fold the AI delays the decline in paw grip endurance (7%) and motor performance (22%) in the G93A mouse [58]. Furthermore, a consequence of vitamin D deficiency is a decrease in the synthesis of the contractile proteins actin and troponin C [64]. Statistical analyses were performed using Statistica 6.0 Windows (version 6.0, StatSoft, Tulsa, OK). Significance was considered at P≤0.05, and trends were considered at 0.05<P≤0.10. Data are presented as means ± standard error of the mean (SEM).

## Results

### Food intake

AI mice had an average food intake of 3.3±0.1 g/d (M = 3.4±0.1 g/d, F = 3.2±0.1 g/d), corresponding to an average vitamin D_3_ intake of 3.3 IU/d (M = 3.4 IU/d, F = 3.2 IU/d) (Supplemental Figure S1A). DEF mice had an average food intake of 3.2±0.1 g/d (M = 3.3±0.1 g/d, F = 3.1±0.1 g/d), corresponding to an average vitamin D_3_ intake of 0.08 IU/d (M = 0.08 IU/d, F = 0.08 IU/d). There were no significant diet- or sex-based differences for food intake.

AI mice had an average food intake corrected for body weight of 167.6±4.1 mg/g b.wt./d (M = 155.7±3.5 mg/g b.wt./d, F = 185.4±6.0 mg/g b.wt./d) (Supplemental Figure S1B). DEF mice had an average food intake corrected for body weight of 165.7±4.1 mg/g b.wt./d (M = 153.0±3.8 mg/g b.wt./d, F = 179.3±5.5 mg/g b.wt./d). There were no significant diet-based differences, however males consumed 15% less food corrected for body weight vs. females (154.4±3.1 mg/g b.wt./d vs. 182.4±3.5 mg/g b.wt./d; P<0.001).

### Body weight

AI mice had an average body weight of 20.2±0.5 g (M = 21.9±0.4 g, F = 17.5±0.3 g) (Supplemental Figure S1C). DEF mice had an average body weight of 19.9±0.5 g (M = 22.1±0.5 g, F = 17.6±0.2 g). There were no significant diet-based differences, however males had 25% higher body weight vs. females (22.0±0.3 g vs. 17.5±0.3 g; P<0.001).

### Ability to move

There were no significant diet-based differences in ability to move over time (Supplemental Figure S2A). Between the sexes, males had 5% (P = 0.047) lower ability to move between age 60–141 d and 15% (P = 0.050) lower ability to move during disease progression vs. females. Over time, ability to move for AI mice was significantly lower than baseline starting at age 119 d (P<0.001); starting at 116 d (P = 0.049) for males and at 123 d for females (P<0.001). For DEF mice, ability to move was significantly lower than baseline starting at age 119 d (P<0.001); starting at 119 d (P = 0.006) for males and at 123 d for females (P<0.001).

### Paw grip endurance (PaGE)

**Figure 1:**
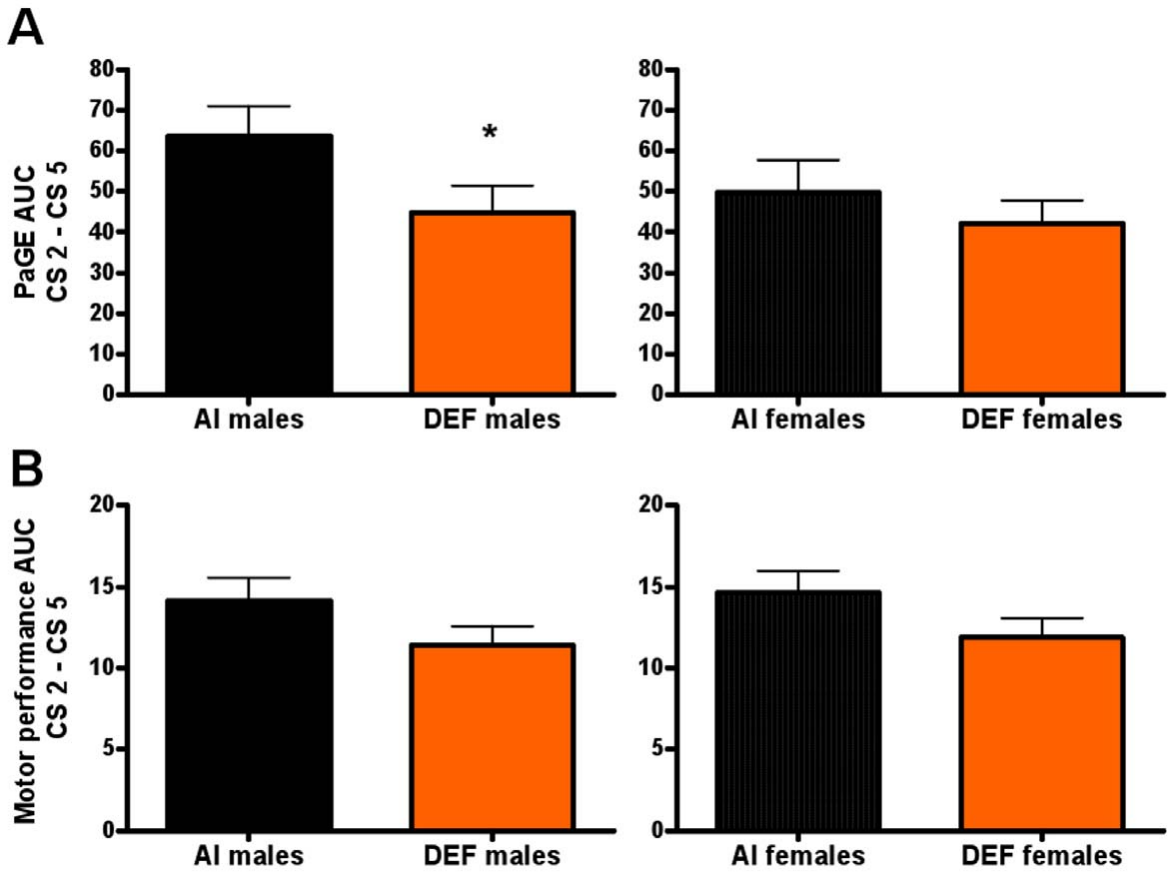
Paw grip endurance and motor performance AUC during disease progression. (A) Paw grip endurance (PaGE) area under the curve (AUC) between CS 2–CS 5 (i.e. during disease progression) and (B) motor performance AUC between CS 2–CS 5 for 31 adequate vitamin D_3_ intake (AI; 1 IU D_3_/g feed; black squares, 19 males; black circles, 12 females) and 29 deficient vitamin D_3_ intake (DEF; 0.025 IU D_3_/g feed; orange squares, 15 males; orange circles, 14 females) G93A mice. (A) During disease progression, DEF mice had 25% (P = 0.022) lower PaGE AUC and 23% (P = 0.028) lower cumulative PaGE vs. AI mice, mainly due to DEF males having 30% (P = 0.039) lower PaGE AUC and 28% (P = 0.047) lower cumulative PaGE vs. AI males; DEF females had a non-significant 14% lower PaGE AUC and 13% lower cumulative PaGE vs. AI females. (B) During disease progression, DEF mice had 19% (P = 0.017) lower motor performance AUC and 18% (P = 0.019) lower cumulative motor performance vs. AI mice. DEF males had 19% (P = 0.073) lower motor performance AUC and 18% (P = 0.077) lower cumulative motor performance, as well DEF females had 19% (P = 0.058) lower motor performance AUC and 18% (P = 0.063) lower cumulative motor performance, vs. their AI counterparts during disease progression. Data presented as means ± SEM. * P = 0.039.

There were no significant diet-based differences in PaGE over time (Supplemental Figure S2B). Between the sexes, males had 13% (P = 0.010) lower PaGE between age 60–141 d, as well as 11% (P = 0.012) and 23% (P = 0.050) lower PaGE prior to disease onset and during disease progression, respectively, vs. females. Over time, PaGE for AI mice was significantly lower than baseline starting at age 97 d (P<0.001); starting at 97 d (P<0.001) for males and at 100 d for females (P = 0.004). For DEF mice, PaGE was significantly lower than baseline starting at age 100 d (P = 0.006); starting at 103 d (P = 0.010) for males and at 100 d for females (P = 0.029). During disease progression, DEF mice had 25% (P = 0.022) lower PaGE AUC and 23% (P = 0.028) lower cumulative scores vs. AI mice, mainly due to DEF males having 30% (P = 0.039) lower PaGE AUC and 28% (P = 0.047) lower cumulative scores vs. AI males; DEF females had a non-significant 14% lower PaGE AUC and 13% lower cumulative scores vs. AI females (Figure 1A). Between the sexes, males had 8% (P = 0.035) lower PaGE AUC and 7% (P = 0.046) lower cumulative scores prior to disease onset vs. females.

### Motor performance

There were no significant diet-based differences in motor performance over time (Supplemental Figure S2C). Between the sexes, males had 18% (P = 0.075) lower motor performance during disease progression vs. females. Over time, motor performance for AI mice was significantly lower than baseline starting at age 110 d (P<0.001); starting at 110 d (P<0.001) for males and at 120 d for females (P<0.001). For DEF mice, motor performance was significantly lower than baseline starting at age 110 d (P = 0.004); starting at 110 d (P = 0.021) for males and at 120 d for females (P<0.001). During disease progression, DEF mice had 19% (P = 0.017) lower motor performance AUC and 18% (P = 0.019) lower cumulative scores vs. AI mice; DEF males had 19% (P = 0.073) lower motor performance AUC and 18% (P = 0.077) lower cumulative scores, as well DEF females had 19% (P = 0.058) lower motor performance AUC and 18% (P = 0.063) lower cumulative scores, vs. their AI counterparts (Figure 1B).

### Clinical score (CS; disease severity)

**Figure 2:**
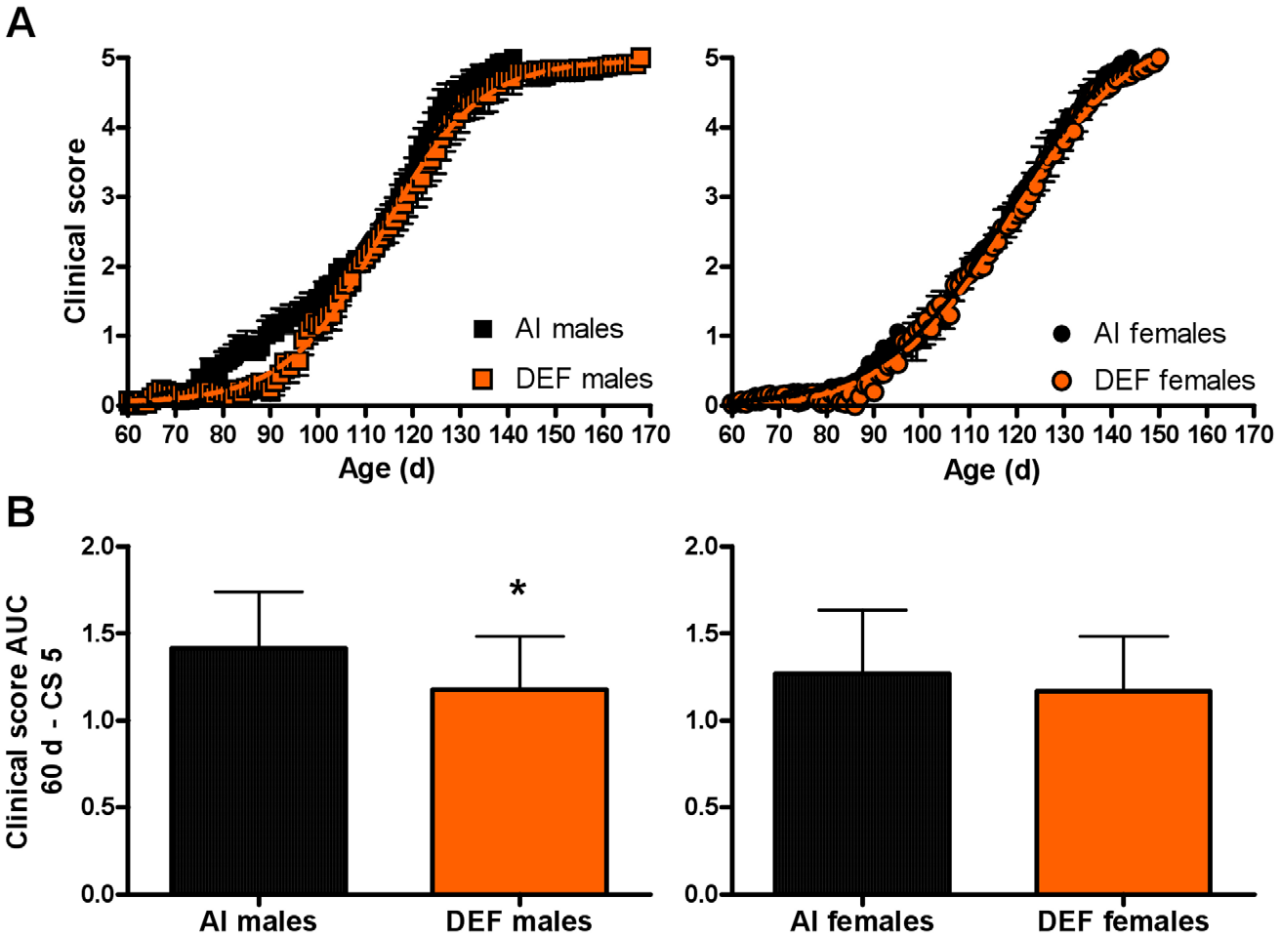
Clinical score over time and AUC. (A) Clinical score (CS) over time and (B) CS area under the curve (AUC) between age 60 d – CS 5 for 31 adequate vitamin D_3_ intake (AI; 1 IU D_3_/g feed; black squares, 19 males; black circles, 12 females) and 29 deficient vitamin D_3_ intake (DEF; 0.025 IU D_3_/g feed; orange squares, 15 males; orange circles, 14 females) G93A mice. (A) Between age 60–141 d, DEF mice had 12% lower CS vs. AI mice (P = 0.029), mainly due to DEF males having 14% lower CS vs. AI males (P = 0.036). Between age 60–105 d (i.e. prior to disease onset), DEF mice had 36% lower CS vs. AI mice (P = 0.016), mainly due to DEF males having 42% lower CS vs. AI males (P = 0.026); DEF females had a non-significant 19% lower CS vs. AI females. (B) Between age 60 d – CS 5, DEF mice had 14% (P = 0.017) lower CS AUC and 13% (P = 0.016) lower cumulative CS vs. AI mice, mainly due to DEF males having 17% (P = 0.039) lower CS AUC and 16% (P = 0.037) lower cumulative CS vs. AI males. Data presented as means ± SEM.

Between age 60–141 d, DEF mice had 12% (P = 0.029) lower CS vs. AI mice (Figure 2A), mainly due to DEF males having 14% (P = 0.036) lower CS vs. AI males. Prior to disease onset, DEF mice had 36% (P = 0.016) lower CS vs. AI mice, mainly due to DEF males having 42% (P = 0.026) lower CS vs. AI males; DEF females had a non-significant 19% lower CS vs. AI females. Between the sexes, males had 15% (P = 0.010) higher CS between age 60–141 d, as well as 38% (P = 0.042) and 11% P = 0.030) higher CS prior to disease onset and during disease progression, respectively, vs. females. Over time, CS for AI mice was significantly higher than baseline starting at age 85 d (P = 0.006); starting at 82 d (P = 0.030) for males and at 95 d for females (P = 0.015). For DEF mice, CS was significantly higher than baseline starting at age 96 d (P = 0.007); starting at 98 d (P = 0.010) for males and at 98 d for females (P = 0.036). Between age 60 d – CS 5, DEF mice had 14% (P = 0.017) lower CS AUC and 13% (P = 0.016) lower cumulative scores vs. AI mice, mainly due to DEF males having 17% (P = 0.039) lower CS AUC and 16% (P = 0.037) lower cumulative scores vs. AI males (Figure 2B).

#### Ability to move vs. CS

DEF mice had 1% (P = 0.027) lower ability to move corrected for CS vs. AI mice, due to DEF females having 3% (P = 0.018) lower ability to move corrected for CS vs. AI females. There were no significant sex-based differences. For AI mice, ability to move corrected for CS was significantly lower than baseline starting at CS 2.25 (P = 0.033); starting at CS 2.5 (P<0.001) for males and at CS 2.5 (P = 0.026) for females. For DEF mice, ability to move corrected for CS was significantly lower than baseline starting at CS 2.5 (P<0.001); starting at CS 2.5 (P = 0.003) for males and at CS 2.5 (P = 0.001) for females. Data followed a sigmoidal relationship for males (AI males, r^2^ = 0.999; DEF males, r^2^ = 0.998; curves were significantly different, P<0.001) and females (AI females, r^2^ = 0.999; DEF females, r^2^ = 0.998; curves were significantly different, P<0.001).

#### PaGE vs. CS

**Figure 3:**
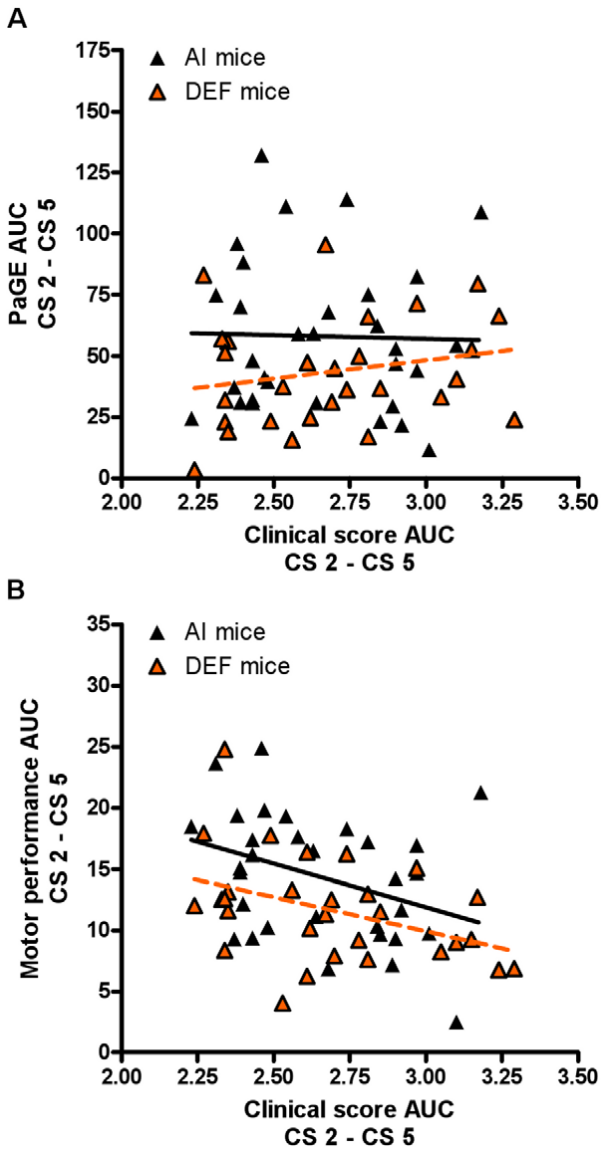
Correlations of paw grip endurance and motor performance vs. clinical score during disease progression. (A) PaGE AUC between vs. CS AUC and (B) motor performance AUC vs. CS AUC during disease progression for 31 adequate vitamin D_3_ intake (AI; 1 IU D_3_/g feed; black triangles) and 29 deficient vitamin D_3_ intake (DEF; 0.025 IU D_3_/g feed; orange triangles) G93A mice. (A) During disease progression, DEF mice (r = 0.213; slope = 14.99; P = 0.268) had a 27% lower PaGE AUC elevation (P = 0.042) for the same CS AUC vs. AI mice (r = −0.026; slope = −3.01; P = 0.891). For AI mice: PaGE AUC CS 2–CS 5 = (66.05±58.23)+[(−3.01±21.82)×(CS AUC CS 2–CS 5)]. For DEF mice: PaGE AUC CS 2–CS 5 = (3.29±35.91)+[(14.99±13.27)×(CS AUC CS 2–CS 5)]. (B) During disease progression, motor performance AUC negatively correlated with CS AUC for both AI (r = −0.359; slope = −7.13; P = 0.047) and DEF (r = −0.404; slope = −5.57; P = 0.030) mice, with DEF mice having 19% lower motor performance elevation vs. AI mice (P = 0.037). For AI mice: motor performance AUC CS 2–CS 5 = (33.29±9.19)+[(−7.13±3.45)×(CS AUC CS 2–CS 5)]. For DEF mice: motor performance AUC CS 2–CS 5 = (26.62±6.56)+[(−5.57±2.42)×(CS AUC CS 2–CS 5)]. Data presented as means ± SEM.

There were no significant diet- or sex-based differences for PaGE corrected for CS. For AI mice, PaGE corrected for CS was significantly lower than baseline starting at CS 1.0 (P = 0.012); starting at CS 1.5 (P = 0.001) for males and at CS 1.5 (P = 0.002) for females. For DEF mice, PaGE corrected for CS was significantly lower than baseline starting at CS 1.0 (P = 0.019); starting at CS 1.75 (P = 0.001) for males and at CS 1.25 (P = 0.016) for females. Data followed a sigmoidal relationship for males (AI males, r^2^ = 0.982; DEF males, r^2^ = 0.992; curves were not significantly different) and females (AI females, r^2^ = 0.992; DEF females, r^2^ = 0.994; curves were not significantly different). During disease progression, DEF mice had a 27% lower PaGE AUC elevation (P = 0.042) for the same CS AUC vs. AI mice (Figure 3A).

#### Motor performance vs. CS

**Figure 4:**
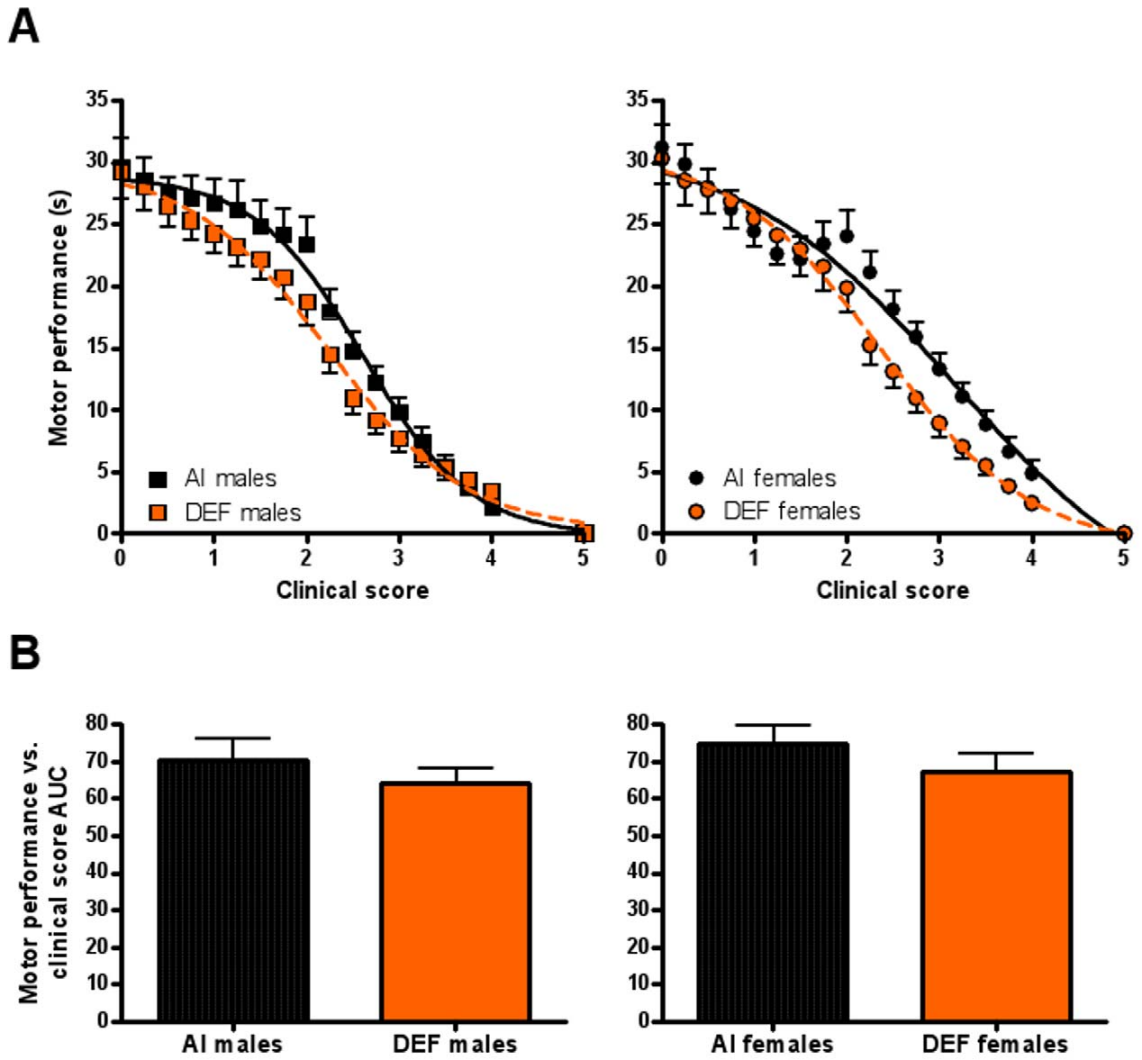
Correlations of motor performance vs. clinical score. (A) motor performance (s) vs. CS and (B) motor performance vs. CS area under the curve (AUC) for 31 adequate vitamin D_3_ intake (AI; 1 IU D_3_/g feed; black squares, 19 males; black circles, 12 females) and 29 deficient vitamin D_3_ intake (DEF; 0.025 IU D_3_/g feed; orange squares, 15 males; orange circles, 14 females) G93A mice. (A) DEF mice had 10% lower motor performance corrected for CS vs. AI mice (P = 0.065). (B) DEF mice had 9% lower motor performance AUC corrected for CS vs. AI mice (P = 0.103). Diet differences were driven by DEF females having 11% lower motor performance corrected for CS vs. AI females (P = 0.103). Data presented as means ± SEM.

DEF mice had 10% (P = 0.065) lower motor performance corrected for CS (Figure 4A) and 9% (P = 0.103) lower motor performance AUC corrected for CS vs. AI mice (Figure 4B). Diet differences were driven by DEF females having 11% (P = 0.103) lower motor performance corrected for CS vs. AI females. There were no significant sex-based differences. For AI mice, motor performance corrected for CS was significantly lower than baseline starting at CS 0.5 (P = 0.038); starting at CS 1.5 (P = 0.013) for males and at CS 0.75 (P = 0.006) for females. For DEF mice, motor performance corrected for CS was significantly lower than baseline starting at CS 0.5 (P = 0.029); starting at CS 0.75 (P = 0.020) for males and at CS 1.0 (P = 0.028) for females. Data followed a sigmoidal relationship for males (AI males, r^2^ = 0.995; DEF males, r^2^ = 0.993; curves were significantly different, P<0.001) and females (AI females, r^2^ = 0.975; DEF females, r^2^ = 0.997; curves were significantly different, P<0.001). During disease progression, motor performance AUC negatively correlated with CS AUC for both AI (r = −0.359; P = 0.047) and DEF (r = −0.404; P = 0.030) mice, with DEF mice having 19% lower motor performance elevation vs. AI mice (P = 0.037) (Figure 3B).

### Disease onset

**Figure 5:**
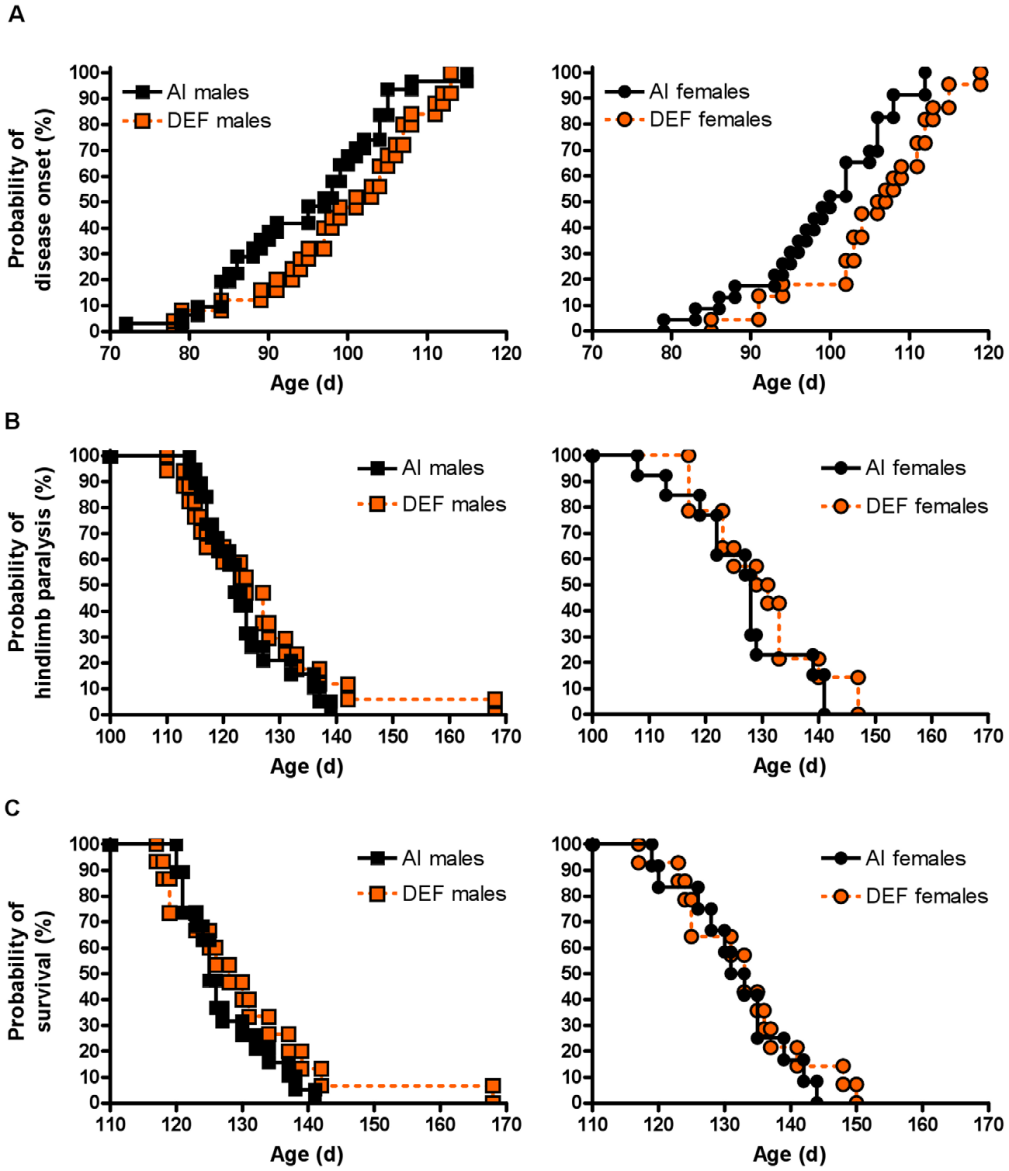
Probability of disease onset, hindlimb paralysis and survival. (A) Probability of disease onset (CS 2; %) for 54 adequate vitamin D_3_ intake (AI; 1 IU D_3_/g feed; black squares, 31 males; black circles, 23 females) and 47 deficient vitamin D_3_ intake (DEF; 0.025 IU D_3_/g feed; orange squares, 25 males; orange circles, 22 females) G93A mice, (B) probability of hindlimb paralysis (CS 4; %) for 32 AI (black squares, 19 males; black circles, 13 females) and 31 DEF (orange squares, 17 males; orange circles, 14 females) G93A mice and (C) probability of survival (CS 5; %) for 31 AI (black squares, 19 males; black circles, 12 females) and 29 DEF (orange squares, 15 males; orange circles, 14 females) G93A mice. (A) DEF mice reached CS 2 at a 43% slower rate vs. AI mice (HR = 0.57; 95% CI: 0.38, 1.74; P = 0.002). DEF males reached CS 2 at a 33% slower rate vs. AI males (HR = 0.67; 95% CI: 0.35, 1.10; P = 0.101). DEF females reached CS 2 at a 50% slower rate vs. AI females (HR = 0.50; 95% CI: 0.21, 0.80; P = 0.009). (B and C) There were no significant diet-based differences in the rate at reaching CS 4 or CS 5.

DEF mice reached disease onset (CS 2) six days later vs. AI mice (102±1 d vs. 96±1 d; P = 0.004). DEF males reached CS 2 five days later vs. AI males (100±2 d vs. 95±2 d; P = 0.073). Similarly, DEF females reached CS 2 six days later vs. AI females (105±2 d vs. 99±2 d; P = 0.021). Using the logrank test, DEF mice reached CS 2 at a 43% slower rate vs. AI mice (HR = 0.57; 95% CI: 0.38, 1.74; P = 0.002) (Figure 5A). DEF males reached CS 2 at a 33% slower rate vs. AI males (HR = 0.67; 95% CI: 0.35, 1.10; P = 0.101). DEF females reached CS 2 at a 50% slower rate vs. AI females (HR = 0.50; 95% CI: 0.21, 0.80; P = 0.009). Between the sexes, males reached CS 2 five days earlier vs. females (97±1 d vs. 102±1 d; P = 0.005). Using the logrank test, males also reached CS 2 at a 58% faster rate vs. females (HR = 1.58; 95% CI: 1.12, 2.60; P = 0.007). In addition, AI males reached CS 2 at a 45% faster rate vs. AI females (HR = 1.45; 95% CI: 0.87, 2.78; P = 0.069), whereas DEF males reached CS 2 at a 74% faster rate vs. DEF females (HR = 1.74; 95% CI: 1.04, 3.68; P = 0.019).

Between 60 d – CS 5, ability to move AUC positively correlated with age at CS 2 for all mice (r = 0.246; P = 0.058), with DEF mice having a 6% delayed disease onset when corrected for ability to move AUC (P = 0.013) vs. AI mice (Supplemental Figure S3A).

Between 60 d – CS 5, PaGE AUC positively correlated with age at CS 2. DEF mice had a 15% delayed disease onset when corrected for PaGE AUC vs. AI mice (P = 0.007) (Supplemental Figure S3B), mainly due to DEF males having a 28% higher elevation vs. AI males (P = 0.043). Prior to disease onset, PaGE AUC positively correlated with age at CS 2. DEF mice had a 16% delayed disease onset when corrected for PaGE AUC vs. AI mice (P = 0.007), mainly due to DEF males having a 25% higher elevation vs. AI males (P = 0.048).

### Hindlimb paralysis and survival

There were no significant differences in the age at hindlimb paralysis (CS 4) between DEF (128±2 d) and AI (125±2 d) mice, or between males (125±2 d) and females (128±2 d). There were no significant diet-based differences in the rate of reaching CS 4 (Figure 5B). Between the sexes, males reached CS 4 at a 42% faster rate vs. females (HR = 1.42; 95% CI: 0.88, 2.53; P = 0.068). In addition, AI males reached CS 4 at a 69% faster rate vs. AI females (HR = 1.69; 95% CI: 0.88, 4.01; P = 0.053), whereas no such difference was observed between DEF males and DEF females.

Ability to move AUC positively correlated with age at CS 4 for all mice between 60 d – CS 5 (r = 0.441; P<0.001) and during disease progression (r = 0.543; P<0.001). During disease progression, DEF mice (r = 0.607; P = 0.001) had a 4% delayed functional paralysis corrected for ability to move AUC vs. AI mice (r = 0.526, P = 0.002) (P = 0.054) (Supplemental Figure S3C), mainly due to DEF males (r = 0.526; P = 0.044) having a 5% higher elevation vs. AI males (r = 0.615; P = 0.005).

There were no significant differences in the age at endpoint (CS 5) between DEF (132±2 d) and AI (129±1 d) mice. There were no significant diet-based differences for the rate at reaching CS 5 (Figure 5C). Between the sexes, males reached CS 5 three days earlier vs. females (129±2 d vs. 132±2 d; P = 0.081). Using the logrank test, males reached CS 5 at a 44% faster rate vs. females (HR = 1.44; 95% CI: 0.88, 2.58; P = 0.067). In addition, AI males reached CS 5 at an 86% faster rate vs. AI females (HR = 1.86; 95% CI: 0.96, 4.42; P = 0.032), whereas no such difference was observed between DEF males and DEF females.

Ability to move AUC positively correlated with age at CS 5 for all mice between 60 d – CS 5 (r = 0.224; P = 0.085) and during disease progression (r = 0.387; P = 0.002).

### Disease Progression (DISPRO)

There were no significant differences in DISPRO between DEF (26±2 d) and AI (30±2 d) mice. During disease progression, ability to move AUC positively correlated with DISPRO for all mice (r = 0.525; P<0.001).

### Tibialis anterior weights

**Figure 6:**
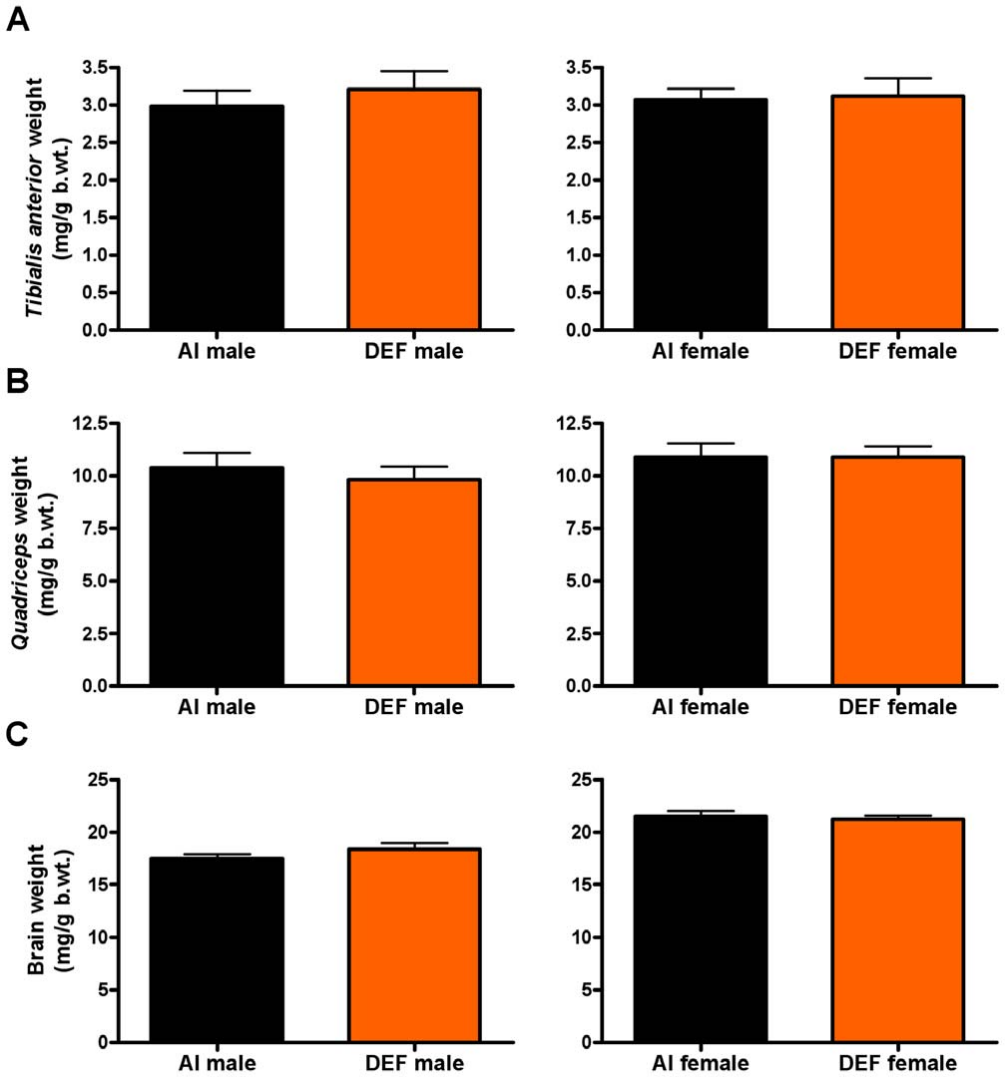
Body weight-adjusted *tibialis anterior*, *quadriceps* and brain weights. Body weight-adjusted (mg/g b.wt.) (A) *tibialis anterior*, (B) *quadriceps* and (C) brain weights for 23 adequate vitamin D_3_ intake (AI; 1 IU D_3_/g feed; black squares, 12 males and 11 females) and 19 deficient vitamin D_3_ intake (DEF; 0.025 IU D_3_/g feed; orange squares, 10 males and 9 females) G93A mice. (A, B and C) There were no significant diet-based differences in body weight-adjusted *tibialis anterior*, *quadriceps* or brain weights. Data presented as means ± SEM.

There were no significant diet-based differences for absolute or body weight-adjusted *tibialis anterior* weights (Figure 6A). Between the sexes, males had 19% heavier absolute *tibialis anterior* weights vs. females (P = 0.012).

**Figure 7:**
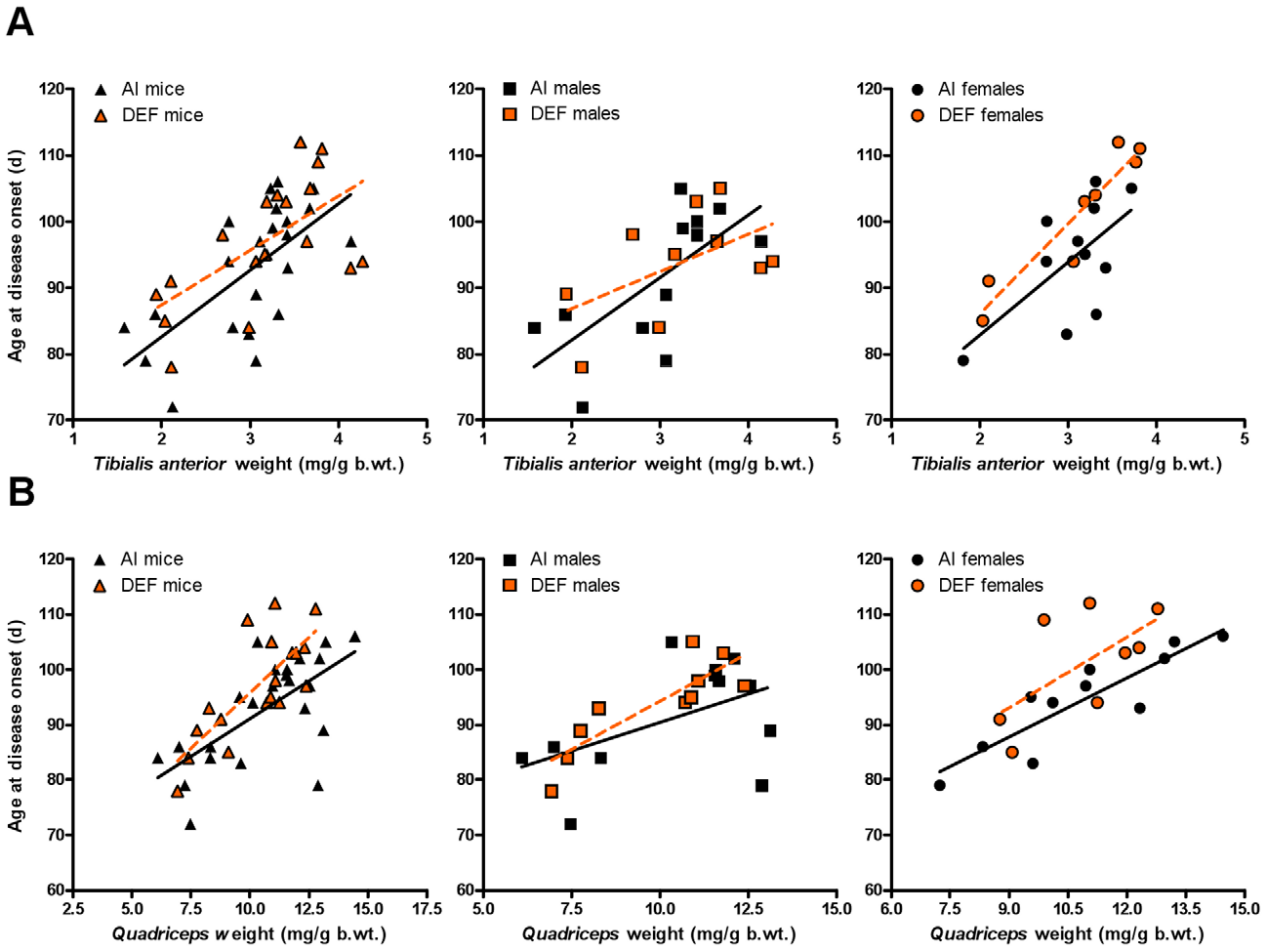
Correlation of disease onset vs. body weight-adjusted *tibialis anterior* and *quadriceps* weights. Age at disease onset (CS 2; d) vs. body weight-adjusted (mg/g b.wt.) (A) *tibialis anterior* weight and (B) *quadriceps* weight for 23 adequate vitamin D_3_ intake (AI; 1 IU D_3_/g feed; black triangles, AI mice; black squares, 12 males; black circles, 11 females) and 18 deficient vitamin D_3_ intake (DEF; 0.025 IU D_3_/g feed; orange triangles, DEF mice; orange squares, 10 males; orange circles, 8 females) G93A mice. (A) Body weight-adjusted *tibialis anterior* weights positively correlated with age at CS 2 for AI (r = 0.662; slope = 10.05; P = 0.001) and DEF mice (r = 0.622; slope = 8.21; P = 0.006). DEF females had a 7% delay in reaching disease onset corrected for TA weight (P = 0.045) vs. AI females. For AI mice: Age at CS 2 (d) = (62.47±7.67)+[(10.05±2.49)×(*tibialis anterior* weight (mg/g b.wt.))]. For DEF mice: Age at CS 2 (d) = (70.96±8.37)+[(8.21±2.58)×(*tibialis anterior* weight (mg/g b.wt.))]. (B) Body weight-adjusted *quadriceps* weights positively correlated with age at CS 2 for AI (r = 0.661; slope = 2.74; P = 0.001) and DEF (r = 0.768; slope = 4.02; P<0.001) mice; DEF mice had a 6% delay in reaching disease onset corrected for quads weight (P = 0.024) vs. AI mice. For AI mice: Age at CS 2 (d) = (63.74±7.36)+[(2.74±0.68)×(*quadriceps* weight (mg/g b.wt.))]. For DEF mice: Age at CS 2 (d) = (55.57±8.76)+[(4.02±0.84)×(*quadriceps* weight (mg/g b.wt.))]. Data presented as means ± SEM.

Body weight-adjusted *tibialis anterior* weights positively correlated with age at CS 2 for AI (r = 0.662; P = 0.001) and DEF (r = 0.622; P = 0.006) mice (Figure 7A). Per 1 mg/g body weight increase in *tibialis anterior* weight, AI and DEF mice experience a 10-d and 8-d delay in disease onset, respectively. Body weight-adjusted *tibialis anterior* weights positively correlated with age at CS 2 for males (AI, r = 0.687, P = 0.014; and DEF, r = 0.537, P = 0.109) and females (AI, r = 0.625, P = 0.040; and DEF, r = 0.941, P = 0.001). Corrected for TA weight, DEF females had a 7% delay in disease onset vs. AI females (P = 0.045).

### *Quadriceps* weights

There were no significant diet-based differences for absolute or body weight-adjusted *quadriceps* weights (Figure 6B). Between the sexes, males had 8% lighter body weight-adjusted *quadriceps* vs. females (P = 0.094).

Body weight-adjusted *quadriceps* weights positively correlated with age at CS 2 for AI (r = 0.661; P = 0.001) and DEF (r = 0.768; P<0.001) mice. Corrected for quads weight, DEF mice had a 6% delay in disease onset vs. AI mice (P = 0.024) (Figure 7B). Per 1 mg/g body weight increase in *quadriceps* weight, AI and DEF mice experience a 3-d and 4-d delay in disease onset, respectively. Body weight-adjusted *quadriceps* weights positively correlated with age at CS 2 for males (AI, r = 0.498, P = 0.100 and DEF, r = 0.850, P = 0.002) and females (AI, r = 0.887, P<0.001; and DEF, r = 0.637, P = 0.089). Corrected for quads weight, DEF females had a 6% delay in disease onset vs. AI females (P = 0.033).

### Brain weights

There were no significant diet-based differences for absolute or body weight-adjusted brain weights (Figure 6C). Between the sexes, males had 17% lighter body weight-adjusted brain weights vs. females (P<0.001).

**Figure 8:**
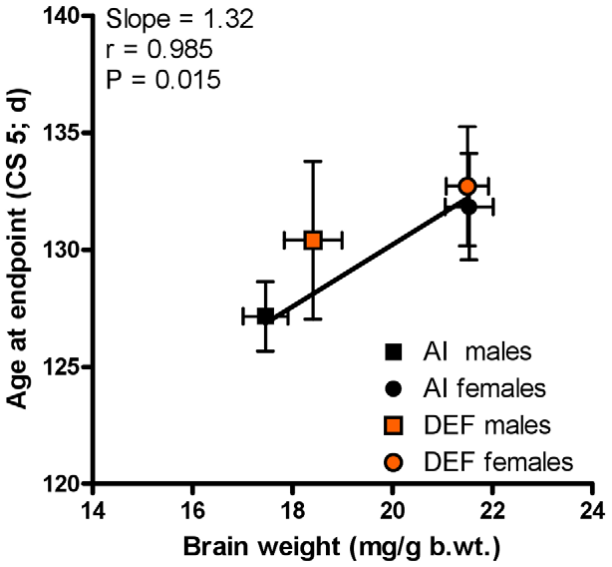
Correlation of group body weight-adjusted brain weights vs. group age at endpoint. The average group body weight-adjusted brain weights (mg/g b.wt.) for 23 adequate vitamin D_3_ intake (AI; 1 IU D_3_/g feed; black squares, 12 males; black circles, 11 females) and 19 deficient vitamin D_3_ intake (DEF; 0.025 IU D_3_/g feed; orange squares, 10 males; orange circles, 9 females) vs. the average group age at endpoint (CS 5; d) for 31 AI (19 males and 12 females) and 29 DEF (15 males and 14 females) G93A mice. Body weight-adjusted brain weights positively correlated with age at CS 5 for all mice (r = 0.985; slope = 1.32; P = 0.015). Age at CS 5 (d) = (103.80±3.19)+[(1.32±0.16)×(brain weights (mg/g b.wt.))]. Data presented as means ± SEM.

Body weight-adjusted brain weights positively correlated with age at CS 5 for all mice (r = 0.985; P = 0.015) (Figure 8). Per 1 mg/g body weight increase in brain weight, mice experience a 1-d delay in CS 5.

## Discussion

We investigated the effects of vitamin D deficiency, via dietary vitamin D_3_ intake equivalent to 2.5% the rodent AI, on functional and disease outcomes in a mouse model of ALS. DEF mice had lower PaGE and motor performance AUC following disease onset, consistent with our previous study showing that vitamin D_3_ supplementation at 10× the AI attenuates the decline in both PaGE and motor performance in G93A mice [58]. Surprisingly, vitamin D deficiency attenuated early disease severity and delayed disease onset in this high-copy G93A mouse model of ALS.

Following disease onset, DEF mice exhibited compromised functional capacity, as depicted by lower PaGE, motor performance and disease severity-adjusted motor performance compared to AI mice. This is consistent with our previous study demonstrating that vitamin D_3_ supplementation at 10× the AI attenuates the decline in PaGE (7%) and motor performance (22%) in the same mouse model of ALS [58]. Moreover, using animal models, vitamin D deficiency affects muscle function *in vivo*. Chicks fed a vitamin D_3_-free diet had 55% lower triceps surae contractile force compared to control chicks fed “starter mash” (8,000 IU vitamin D_3_ plus 21.5 g calcium per kg feed) [65]. Providing additional calcium (31 g/kg feed) to the vitamin D_3_-free diet increased force of contraction by 48% compared to the vitamin D_3_-free diet, albeit remained 33% lower than controls, indicating a role for vitamin D in skeletal muscle independent of calcium. In a separate study, chicks initially fed a vitamin D_3_-free diet then supplemented with 80 IU/d vitamin D_3_ showed higher concentrations of the contractile proteins actin and troponin C compared to chicks maintained on the vitamin D_3_-free diet [64]. In addition to compromising the synthesis of contractile protein, vitamin D deficiency impairs the ability of the sarcoplasmic reticulum to re-uptake calcium following a muscle contraction resulting in a delay of muscle relaxation [65], [66], and compromising subsequent muscle contractions and performance [67]. Accordingly, human studies have linked low vitamin D status to reduced thigh strength [68] and sit-to-stand performance [69]. In 4,100 men and women, subjects in the highest quintile of serum calcidiol (86–400 nmol/L) had higher sit-to-stand test scores compared to those in the lowest quintile (9–43 nmol/L) [69]. In a randomized control trial, 113 institutionalized elderly women (age, ≥70 y) supplemented with either a conventional (880 IU/d) or high (1,600 IU/d) dose of vitamin D_3_ showed a modest increase from baseline in dynamic muscle strength (+8% and +5%, respectively) after 6 mo of supplementation [70]. Although the authors reported that 60% of the participants had initial serum calcidiol levels below 50 nmol/L, baseline average serum calcidiol concentrations were 51.3 nmol/L for the conventional group and 53.6 nmol/L for the high vitamin D_3_ group. More robust changes may have been observed had the participants had lower baseline calcidiol levels. Indeed, whether vitamin D supplementation conclusively affects muscle function is not clear. A recent meta analysis of 17 randomized control trials found that patients with baseline calcidiol levels of ≤25 nmol/L showed an improvement in proximal lower limb muscle strength (standard mean difference = 3.52; 95% CI: 2.18, 4.85) following vitamin D supplementation (cholecalciferol, ergocalciferol, or calcitriol), but not in adults with calcidiol levels >25 nmol/L [71], indicating that muscle may be most compromised under vitamin D deficient conditions and hence more responsive to improved vitamin D status. A recent study found no evidence of the VDR in muscle [28], hence vitamin D and/or its metabolites may exact their effects in skeletal muscle through non-genomic pathways.

*In vitro*, calcitriol is neuroprotective [33], [72]. Pretreatment of cultured rat cortical neurons with 10 nM and 100 nM calcitriol, followed by a glutamate insult, improves neuron survival by ∼17% and ∼50%, respectively [33]. The higher dose also increased VDR mRNA by 3 fold compared to a calcitriol-free control [33]. In addition, co-administration of calcitriol and progesterone improved rat neuron survival by 44% compared to progesterone alone following a glutamate insult [72]. This may be due to several mechanisms: reduction in L-type voltage sensitive Ca^2+^ channel (LVSCC) activity and LVSCC subunit mRNA, with a subsequent decrease in the influx of intracellular Ca^2+^ via LVSCCs (a mechanism implicated in neurotoxicity) [73], [74]; increase in glial cell line derived neurotrophic factor (GDNF) by ∼2 fold [38], promoting motor neuron survival [39]; increases in nerve growth factor (NGF) mRNA [75] and protein [76], increasing neuron survival [77], [78] and inducing neuronal proliferation [78], [79] and differentiation [80]; increases in neuronal filament proteins by ∼55–80% [microtubule-associated protein 2 (MAP2) and growth associated protein 43 (GAP-43)] and synaptosomal protein (Synapsin-1) by ∼65–90%, promoting neuronal cell differentiation and maturation [33].

Paradoxically, dietary vitamin D_3_ restriction mitigated early disease severity and delayed disease onset. In contrast to the present study, in a mouse model of multiple sclerosis (EAE), mice fed a vitamin D_3_-free diet reached disease onset 43% earlier compared to mice fed a diet supplemented with 20 ng/d of calcitriol [44]. Pregnant mothers were fed a diet devoid of vitamin D beginning two weeks into pregnancy and the offspring were subsequently placed on either the same vitamin D-free diet or a calcitriol-supplemented diet at weaning. Weanling offspring given the vitamin D-free diet were predisposed to vitamin D deficiency, as reflected through their very low serum calcitriol concentrations (<13–34 pmol/L) compared to the calcitriol-supplemented offspring (99–169 pmol/L) at 6 wk of age. Conversely, using the same EAE mouse model, Fernandes de Abreu et al [45] as well as Deluca and Plum [46] found that vitamin D deficiency delays disease onset and lowers disease severity compared to EAE controls. In the Fernandes de Abreu et al study, there was a significant delay in disease onset (17–34%) and lower peak disease severity (61%) in offspring born to mothers consuming a normo-calcemic vitamin D_3_-free diet but were themselves placed on the control diet following birth, as compared to control mice (both mother and offspring fed the control diet containing 1,500 IU vitamin D_3_/kg feed) [45]. The authors concluded that the vitamin D deficient environment during gestation induced higher sensitivity to vitamin D in pups, as evidenced by an upregulation of VDR mRNA at birth by ∼3.7 fold. The subsequent switch to a normal diet upon birth was akin to supplementing control mice with high doses of vitamin D_3_, hence resulting in the observed changes in disease onset, disease severity and immune response (66% lower TNF-α mRNA at 30 d post-immunization vs. controls). The mechanisms proposed by the authors center around an upregulation of the VDR, however we propose a simpler mechanism explaining the observed attenuation in disease severity prior to CS 2 and delay in disease onset in the DEF mice in the present study. Following dietary vitamin D_3_ restriction, serum calcidiol concentrations would gradually decrease over time to well below those in the control group, with a concomitant compensatory increase in serum calcitriol concentrations. This is supported by studies showing greater serum calcitriol concentrations in rodents with insufficient dietary intake of vitamin D_3_ or suboptimal serum calcidiol [81]–[83]. However, under prolonged dietary vitamin D_3_ restriction or severely compromised vitamin D status, physiological stores of vitamin D_3_ would be exhausted [81], [83], as reflected by calcidiol concentrations too low to maintain high calcitriol levels even in the presence of a combination of enhanced calcitriol synthesis (CYP27B1) together with reduced calcitriol breakdown (CYP24A1) [81]. Based on this, supranormal calcitriol concentrations would coincide with the period prior to disease onset predicated on both the half-life of calcidiol and calcitriol clearance [81], [84], with a subsequent precipitous decline in calcitriol upon exhaustion of vitamin D_3_ stores and circulating calcidiol as evidenced by the enhanced rate of increase in CS peri- and post- disease onset (age, ≥90 d; Figure 2A). This is corroborated by Fleet et al [81] demonstrating that mice consuming 0.050 IU D_3_/g feed diet had serum calcidiol levels ∼23% those on 1 IU D_3_/g feed diet (equivalent to the AI diet in the current study), and those consuming 0.025 IU D_3_/g feed diet (equivalent to the DEF diet in the current study) had serum calcitriol levels less than 50% those on the AI diet following 11 wk of dietary intervention (age, 98 d) [81]. If this is the case, why did DEF mice not exhibit improved functional outcomes compared to AI mice? Calcitriol is the most active form of vitamin D and possesses neuroprotective properties [33], [72]. Calcidiol has some similar, but less robust, physiological and cellular roles contingent upon specific tissues, and induces gene expression separate from calcitriol [85]. Taken together, we hypothesize that in the presence of low calcidiol the supranormal concentrations of calcitriol rescued the muscle from declining in function, but the subsequent severe vitamin D deficient environment peri- and post-CS 2 compromised muscle function. The compensatory increase in calcitriol pre-disease onset may have also improved immune function. *In vitro*, calcitriol has shown to moderate levels of ROS, NF-kB, IL-1β, IL-2, IL-6, IL-8, and TNF-α [34], [36], [37]. Moreover, calcitriol activates T cells which have neuroprotective effects and slow disease progression in an animal model of ALS [86]–[88]. Post-disease onset, however, the immune system would be deprived of the anti-oxidant effects of calcitriol. This is corroborated by our previous observation that vitamin D_3_ supplementation at 10× the AI attenuated the decline in both PaGE and motor performance in the G93A mouse model of ALS [58]. Based on this, we hypothesize that both calcidiol and calcitriol have tissue-specific functions [85], [89].

Between the sexes, males had lower ability to move, PaGE and motor performance compared to females. Males also had a higher clinical score and hastened disease onset, hindlimb paralysis and endpoint compared to females. Interestingly, vitamin D deficiency attenuated this sexual dichotomy; AI males reached CS 4 and CS 5 at a faster rate compared to AI females, whereas no such difference was observed between DEF males and DEF females. In addition, males were more responsive to vitamin D deficiency; diet differences were mainly driven by DEF males having significantly lower PaGE following disease onset and lower early disease severity. Females were more resistant to a sharp reduction in vitamin D_3_ intake, possibly due to the interaction between estrogen and vitamin D metabolites. In a mouse model of EAE, female mice supplemented with vitamin D_3_ (40 IU/d) had 67% higher calcitriol, 75% lower calcitriol-deactivating enzyme CYP24A1 mRNA, and 65% higher CYP27B1: CYP24A1 mRNA ratio (enzyme transcript ratio coding for calcitriol synthesis:deactivation) in the spinal cord compared to supplemented males [30]. The ability of females to synthesize more calcitriol in the CNS compared to males may have predisposed the G93A females to relatively higher basal CNS calcitriol, levels attained by the G93A males, albeit transiently, only under vitamin D deficiency. Accordingly, as vitamin D_3_ intake decreases below 0.40 IU D_3_/g feed, CYP24A1 mRNA decreases, and at very low levels of vitamin D_3_ intake (below 0.05 IU D_3_/g feed) CYP27B1 mRNA increases [81]. It is possible that restricting dietary vitamin D_3_ may not effect significant increases in CNS calcitriol in females above their heightened basal levels.

During disease progression, ability to move was positively correlated with age at hindlimb paralysis. As well, both AI and DEF mice had positive correlations between body weight-adjusted *tibialis anterior* and *quadriceps* weights with age at CS 2, indicating that improvements in muscle mass and function may delay disease onset and paralysis. This is corroborated by previous research showing that G93A mice supplemented with 2% creatine monohydrate (CrM) had 25% greater *extensor digitorum longus* weight (mg/g b.wt.) [90] and a significant 7% delay in disease onset (∼12 d) compared to non-supplemented G93A mice [91]. Furthermore, supplementing human ALS patients with 20 g/d of CrM for 7 d increased maximal voluntary isometric muscular contraction in knee extensors (70%) and elbow flexors (53%) compared to pre-treatment values [92]. These results emphasize the need for clinicians to focus on increasing muscle mass and function of ALS patients to improve prognosis.

In spite of some limitations, the underlying pathophysiologies common to both the G93A mouse model and ALS patients makes extrapolating to humans plausible [5], [9]. At 25 d of age, when dietary intervention commenced, G93A mice did not exhibit the ALS phenotype. As well, the first mean group disease onset was reached 70 d following commencement of intervention (at 95 d of age). In contrast, the time period between the first appearance of ALS symptoms and clinical diagnosis varies widely in ALS patients, ranging between 6–38 mo [93]–[95]. Accordingly, ALS patients are prescribed nutritional or pharmaceutical therapeutics well after the initiation of overt signs and symptoms, when CNS damage has become irreversible. Would manipulating dietary vitamin D_3_ intake in ALS patients improve prognosis? There is a dearth of information on the vitamin D status of ALS patients. One study found that serum calcitriol was in the low-normal range (29–65 pmol/l), but did not report serum calcidiol [96]. The observed attenuation in disease severity and delayed disease onset in DEF mice in the present study may be counterintuitive. Physiologically, these improvements in disease outcomes coincide with the adaptive compensatory increase in calcitriol simultaneous with reductions in calcidiol following dietary vitamin D_3_ restriction [81]. In evidence, DEF mice exhibited compromised functional capacity following disease onset, congruent with exhausted stores of endogenous vitamin D_3_ and diminished synthesis of calcidiol and calcitriol, predicated on the half-lives of calcidiol and calcitriol as well as the accelerated progress of disease and short life span characteristic of this high-copy G93A mouse model. Furthermore, dietary vitamin D_3_ at 10 fold the AI improves functional capacity in the same mouse model [58]. To address the current gaps in our understanding, future studies should: ascertain the effects of prolonged vitamin D deficiency on functional and disease outcomes in the low-copy G93A mouse model of ALS, a model with a disease onset of 195–205 d and lifespan of 237–250 d of age [97]–[99]; measure the temporal changes in serum, skeletal muscle and CNS vitamin D metabolites (specifically, calcidiol and calcitriol) following dietary vitamin D_3_ restriction in G93A rodent models; investigate the use of calcitriol as a treatment in G93A mice; and provide rodent models of ALS with vitamin D deficient and supplemented diets concurrent with disease onset.

In conclusion, the present study demonstrates that vitamin D deficiency differentially affects functional and disease outcomes. Dietary vitamin D_3_ restriction (0.025 IU/g feed) decreases PaGE and motor performance, improves early disease severity and delays disease onset compared to a diet with adequate vitamin D_3_ (1 IU/g feed) in the high-copy G93A mouse model of ALS. We hypothesize that the attenuation of early disease severity and the delay in disease onset may have been due to a transient compensatory increase in calcitriol under conditions of decreasing calcidiol concentrations, whereas the reduced functional performance may be due to changes in intramuscular calcium regulation and a decrease in contractile protein synthesis. Based on *in vitro* [100] and *in vivo* studies [85], [89], we hypothesize that our results may also suggest a possible tissue-specific effect of vitamin D and its metabolites in the CNS as compared with skeletal muscle. Further research in this animal model is needed prior to translating these results to humans. Identifying the mechanism(s) responsible for the observed improvement in early disease severity and delay in disease onset may help deliver novel therapies to ALS patients.

## Supporting Information

Figure S1 **Food intake, food intake corrected for body weight and body weight over time.** (A) Food intake (g), (B) food intake corrected for body weight (mg/g b.wt./d) and (C) body weight (g) for 31 adequate vitamin D_3_ intake (AI; 1 IU D_3_/g feed; black squares, 19 males; black circles, 12 females) and 29 deficient vitamin D_3_ intake (DEF; 0.025 IU D_3_/g feed; orange squares, 15 males; orange circles, 14 females) G93A mice. (A, B and C) There were no significant diet-based differences in food intake, food intake corrected for body weight or body weight over time. Data presented as means ± SEM.
(TIF)

Figure S2 **Ability to move, paw grip endurance and motor performance over time.** (A) Ability to move, (B) Paw grip endurance (PaGE; s) and (C) motor performance (s) over time for 31 adequate vitamin D_3_ intake (AI; 1 IU D_3_/g feed; black squares, 19 males; black circles, 12 females) and 29 deficient vitamin D_3_ intake (DEF; 0.025 IU D_3_/g feed; orange squares, 15 males; orange circles, 14 females) G93A mice. (A, B and C) There were no significant diet-based differences in ability to move, PaGE or motor performance over time. Data presented as means ± SEM.
(TIF)

Figure S3 **Correlations of disease and functional outcomes.** Age at disease onset (CS 2; d) vs. (A) Ability to move area under the curve (AUC) and (B) paw grip endurance (PaGE) AUC between age 60 d – CS 5, and (C) age at hindlimb paralysis (CS 4; d) vs. ability to move AUC during disease progression for 31 adequate vitamin D_3_ intake (AI; 1 IU D_3_/g feed; black triangles) and 29 deficient vitamin D_3_ intake (DEF; 0.025 IU D_3_/g feed; orange triangles) G93A mice. (A) Between 60 d – CS 5, DEF mice (r = 0.185; slope = 16.12; P = 0.338) had a 6% delayed disease onset when corrected for ability to move AUC (P = 0.013) vs. AI mice (r = 0.229; slope = 19.02; P = 0.215). (B) Between 60 d – CS 5, DEF mice (r = 0.311; slope = 0.087; P = 0.100) had a 15% delayed disease onset when corrected for PaGE AUC (P = 0.007) vs. AI mice (r = 0.175; slope = 0.067; P = 0.347), mainly due to DEF males (r = 0.375; slope = 0.087; P = 0.169) having a 28% higher elevation (P = 0.043) vs. AI males (r = 0.092; slope = 0.031; P = 0.708). (C) During disease progression, DEF mice (r = 0.607; slope = 21.76; P = 0.001) had a 4% delayed functional paralysis corrected for ability to move AUC (P = 0.054) vs. AI mice (r = 0.526, slope = 13.48; P = 0.002), mainly due to DEF males (r = 0.526; slope = 20.55; P = 0.044) having a 5% higher elevation (P = 0.104) vs. AI males (r = 0.615; slope = 15.09; P = 0.005). Data presented as means ± SEM.
(TIF)
