# Supplementary figures and images for: Vitamin D3 Deficiency Differentially Affects Functional and Disease Outcomes in the G93A Mouse Model of Amyotrophic Lateral Sclerosis
Source: PLoS One. 2011 Dec 27;6(12):e29354. doi: 10.1371/journal.pone.0029354 (PMC3246470; doi:10.1371/journal.pone.0029354)

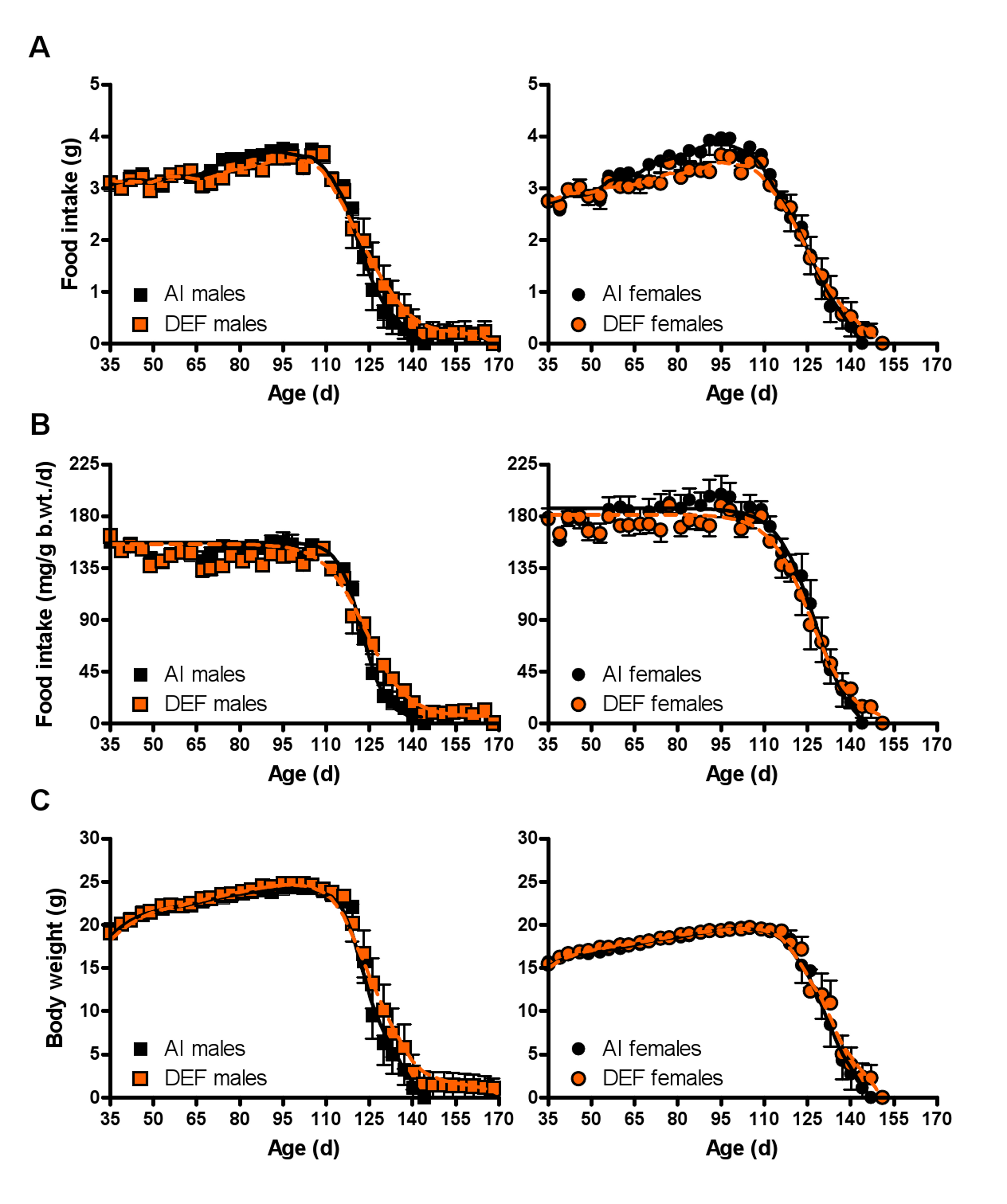

Supplement: Figure S1 — Food intake, food intake corrected for body weight and body weight over time. (A) Food intake (g), (B) food intake corrected for body weight (mg/g b.wt./d) and (C) body weight (g) for 31 adequate vitamin D3 intake (AI; 1 IU D3/g feed; black squares, 19 males; black circles, 12 females) and 29 deficient vitamin D3 intake (DEF; 0.025 IU D3/g feed; orange squares, 15 males; orange circles, 14 females) G93A mice. (A, B and C) There were no significant diet-based differences in food intake, food intake corrected for body weight or body weight over time. Data presented as means ± SEM. (TIF) [file pone.0029354.s001.tif]

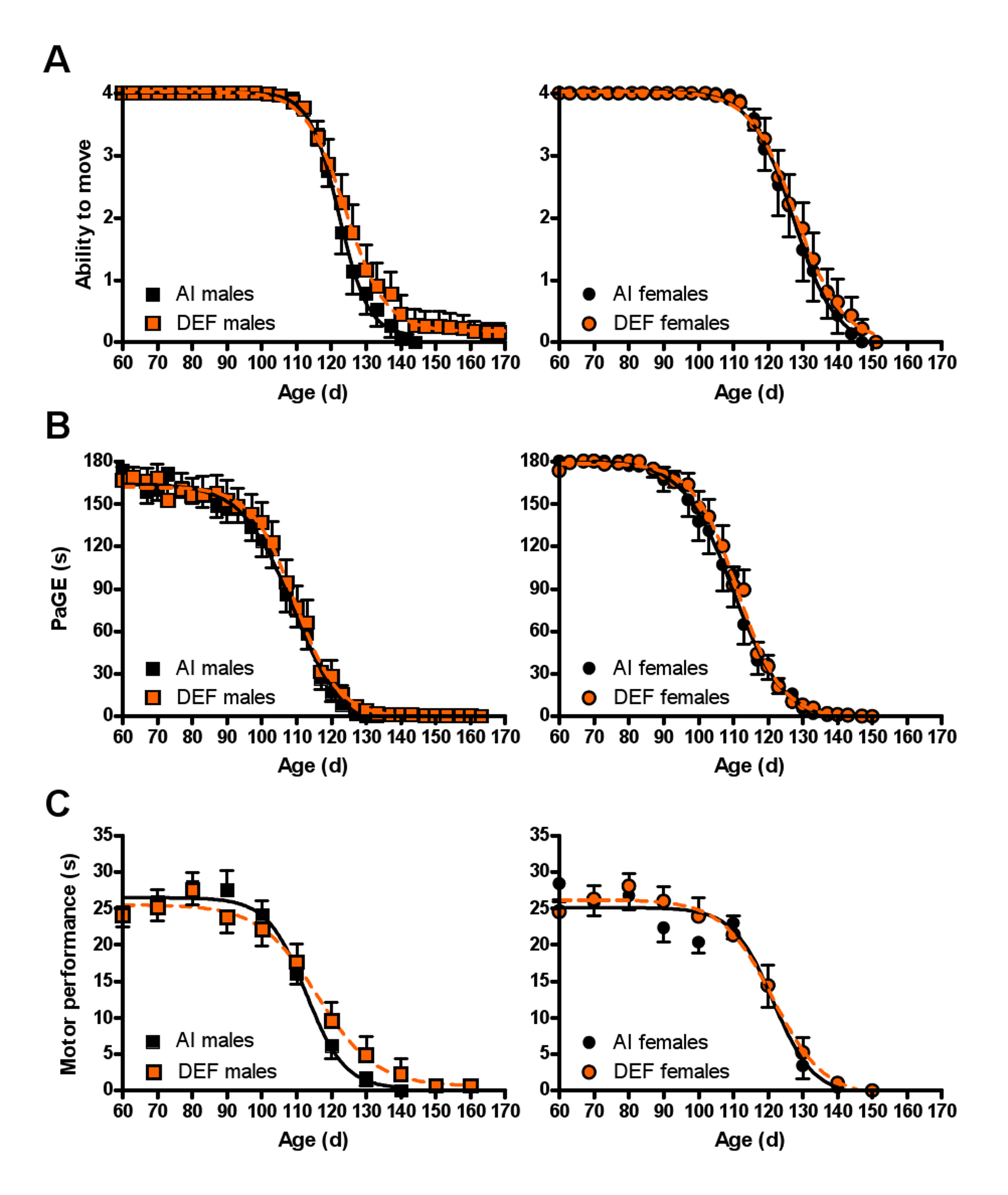

Supplement: Figure S2 — Ability to move, paw grip endurance and motor performance over time. (A) Ability to move, (B) Paw grip endurance (PaGE; s) and (C) motor performance (s) over time for 31 adequate vitamin D3 intake (AI; 1 IU D3/g feed; black squares, 19 males; black circles, 12 females) and 29 deficient vitamin D3 intake (DEF; 0.025 IU D3/g feed; orange squares, 15 males; orange circles, 14 females) G93A mice. (A, B and C) There were no significant diet-based differences in ability to move, PaGE or motor performance over time. Data presented as means ± SEM. (TIF) [file pone.0029354.s002.tif]

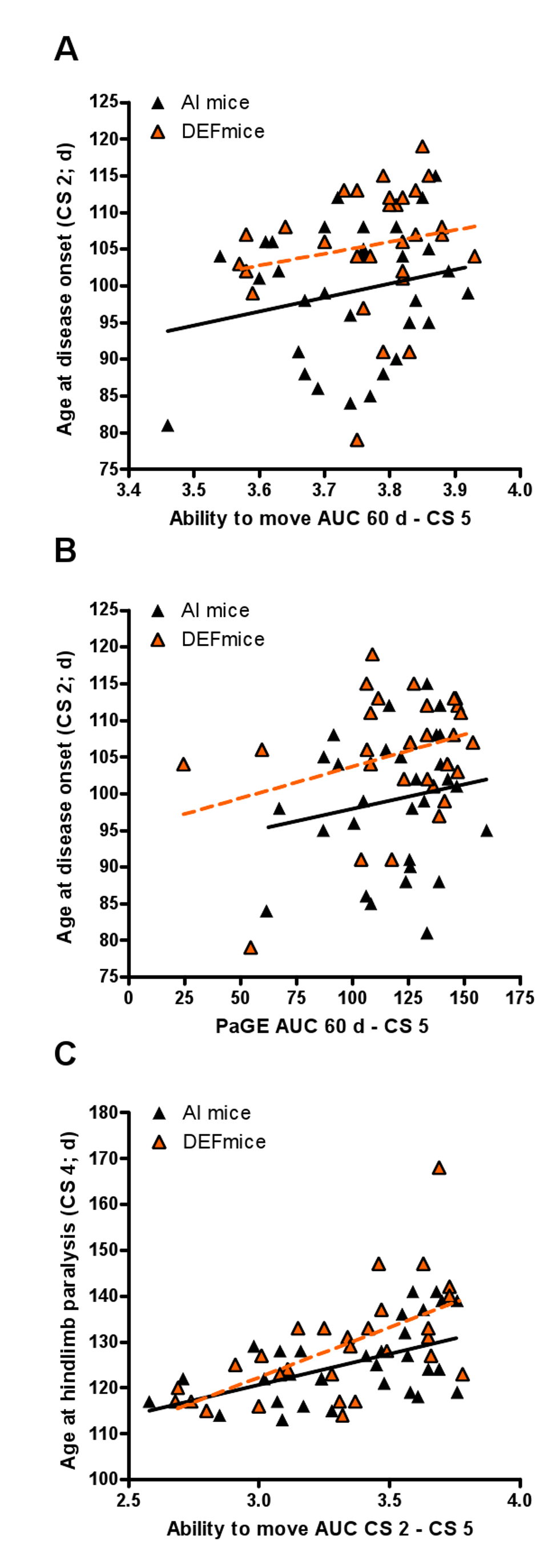

Supplement: Figure S3 — Correlations of disease and functional outcomes. Age at disease onset (CS 2; d) vs. (A) Ability to move area under the curve (AUC) and (B) paw grip endurance (PaGE) AUC between age 60 d – CS 5, and (C) age at hindlimb paralysis (CS 4; d) vs. ability to move AUC during disease progression for 31 adequate vitamin D3 intake (AI; 1 IU D3/g feed; black triangles) and 29 deficient vitamin D3 intake (DEF; 0.025 IU D3/g feed; orange triangles) G93A mice. (A) Between 60 d – CS 5, DEF mice (r = 0.185; slope = 16.12; P = 0.338) had a 6% delayed disease onset when corrected for ability to move AUC (P = 0.013) vs. AI mice (r = 0.229; slope = 19.02; P = 0.215). (B) Between 60 d – CS 5, DEF mice (r = 0.311; slope = 0.087; P = 0.100) had a 15% delayed disease onset when corrected for PaGE AUC (P = 0.007) vs. AI mice (r = 0.175; slope = 0.067; P = 0.347), mainly due to DEF males (r = 0.375; slope = 0.087; P = 0.169) having a 28% higher elevation (P = 0.043) vs. AI males (r = 0.092; slope = 0.031; P = 0.708). (C) During disease progression, DEF mice (r = 0.607; slope = 21.76; P = 0.001) had a 4% delayed functional paralysis corrected for ability to move AUC (P = 0.054) vs. AI mice (r = 0.526, slope = 13.48; P = 0.002), mainly due to DEF males (r = 0.526; slope = 20.55; P = 0.044) having a 5% higher elevation (P = 0.104) vs. AI males (r = 0.615; slope = 15.09; P = 0.005). Data presented as means ± SEM. (TIF) [file pone.0029354.s003.tif]
